# Synergistic Effect of Hybridized Cellulose Nanocrystals and Organically Modified Montmorillonite on κ-Carrageenan Bionanocomposites

**DOI:** 10.3390/nano8110874

**Published:** 2018-10-24

**Authors:** Siti Zarina Zakuwan, Ishak Ahmad

**Affiliations:** School of Chemical Sciences and Food Technology, Faculty of Science and Technology, Universiti Kebangsaan Malaysia, 43600 Bangi Selangor, Malaysia; p90319@siswa.ukm.edu.my

**Keywords:** hybrid composites, nanocomposites, mechanical properties, synergism, nanoparticles

## Abstract

The synergistic effect of using κ-carrageenan bionanocomposites with the hybridization of cellulose nanocrystals (CNCs) and organically modified montmorillonite (OMMT) reinforcements was studied. The effects of different reinforcements and filler contents were evaluated through mechanical testing, and morphological and water uptake properties. The tensile strength and Young’s modulus of both bionanocomposites increased with filler loading and optimized at 4%. OMMT incorporation into the κ-carrageenan/CNCs bionanocomposites resulted in further mechanical property improvement with an optimum ratio of 1:1 (CNCs:OMMT) while maintaining high film transparency. X-ray diffraction and morphological analyses revealed that intercalation occurred between the κ-carrageenan bionanocomposite matrix and OMMT. The water uptake of the κ-carrageenan bionanocomposites was significantly reduced by the addition of both CNCs and OMMT. The enhancements in the mechanical properties and performance of the hybrid bionanocomposite indicate compatibility among the reinforcement, biopolymer, and well-dispersed nanoparticles. This renders the hybrid CNC/OMMT/κ-carrageenan nanocomposites extremely promising for food packaging applications.

## 1. Introduction

This research focuses on the hybridization of polysaccharide, nanocellulose, and modified nanoclay to overcome the problem of low strength performance which is characteristic of bioplastics, by improving the mechanical properties. Polysaccharide from plants, such as κ-carrageenan (κ-carr) extracted from red seaweed (*Eucheuma cottonii*), naturally contains one negative charge per-disaccharide unit which can form a strong and rigid gel; further, it has great potential as a film-forming material. Carrageenan is unique in that it possesses both hydrophilic and ionic properties the latter influencing the former, which provides interesting possibilities for applications. More specifically, κ-carr is promising because of its excellent properties, including nontoxicity, biocompatibility, and biodegradability, which have enabled their wide use in the food, cosmetics, and pharmaceuticals industries. However, κ-carr films have exhibited relatively poor performance because of brittleness and barrier properties which limit their use in applications.

Bio-based or natural organic fillers, such as cellulose nanocrystals (CNCs) that have been isolated from cellulose, have attracted considerable interest within the biopolymer community owing to cellulose’s renewable nature. CNCs extracted from kenaf fiber (*Hibiscus cannabinus*) are particularly interesting since this fiber is readily available, which renders it cost effective. Natural nanofillers’ other benefits include high specific strengths and moduli, good adhesion, relatively high aspect ratio, lighter weight, and biodegradability [1]. These properties render CNCs an attractive nanomaterial class for industrial packaging and result in their being continuously studied to determine innovative solutions for efficient and sustainable systems [2,3,4].

Among various inorganic nanoparticles, organoclays are cheaper since they are produced from readily available natural sources and are commercially available. Organically modified montmorillonite (OMMT) layered clay, with a negatively charged surface, compensates with an exchangeable cation. The polarity of OMMT is different from that of the biopolymer, with parallel layers linked together by weak electrostatic forces. The long organic chains with positively charged ends inside the OMMT gallery result in an increase in the gallery height. This facilitates polymer-chain intercalation [5]. Clay has received considerable attention in the nanocomposite field for its ability to enhance biopolymer performance, and provide significant enhancement at low filler loadings, high strength, and stiffness with a high aspect ratio for every single platelet, and excellent barrier properties [6,7,8]. Another factor which could also affect the filler matrix interaction in polymer nanocomposites is the surface porosity of nanofillers. Akhlaghi et al., reported that heat-treated ZnO particles dispersed better than ZnO nanoparticles in acrylonitrile butadiene rubber (NBR) and promoted the curing of NBR [9].

Hybrid bionanocomposites have been used in diverse interesting applications in different fields due to the properties that arise from the synergistic effects that occur in the reinforcement region [10]. Growing concerns over environmental issues and the high demand for advanced polymeric materials with balanced properties have led to hybrid composite development by combining natural fiber/natural fiber, natural fiber/nanofiller inorganic or natural fiber/nanofiller from organic sources as an alternative to synthetic fibers [11,12,13]. Previous research on the hybridization of nanofiller and natural fiber in the matrix demonstrate improved mechanical properties and a reduction in water absorption properties [14]. There are only few studies on the utilization of hybrid nanofillers in a bio-nanocomposite. Petersson and Oksman [15] compared layered silicates and microcrystalline cellulose as a reinforcement in polylactic acid nanocomposites. Recent studies on hybrid nanoclay in an alginate hydrogel containing calcium ions reported that biocompatibility enables the formation of a better network between alginate and nanoclay due to ionic interaction with ion exchange between the matrix and filler. The stability of nanoclays in the matrix dispersion is controlled by several parameters such as the surface charge and particle size, which highlight their potential in the medical field [16,17]. Bendahou et al., [18] investigated the synergistic effect of montmorillonite (MMT) and CNCs on the mechanical and barrier properties of natural rubber composites. However, the comparison of the effect of OMMT and CNCs on the κ-carr films’ physicochemical properties, which have SO_3_^−^ groups, was not investigated; thus, the current research is the first comparative study in this area. Addition of hybrid reinforcement CNC and OMMT in κ-carr polymer is expected to improve composite-polymer interfacial interaction and improve the properties the properties of the bionanocomposite.

This paper discusses the development of novel nanohybrid inorganic nanoclay/organic nanofillers based on the κ-carr biopolymer in a one-step/in situ process and the synergistic effects of the nanohybrid on the mechanical and barrier properties.

## 2. Materials and Methods

### 2.1. Materials

The κ-carr was obtained from TACARA Natural Carrageenan (Sabah, Malaysia) with a moisture content <12%, pH 9–10. Raw kenaf fibers were supplied by Kenaf Fiber Industry Sdn. Bhd. (Kelantan, Malaysia). OMMT with 25–30 wt% methyl dihydroxyethyl hydrogenated tallow ammonium (Nanocor®, AMCOL International, Hoffman Estates, IL, USA). Sigma-Aldrich (St. Louis, MO, USA), sulfuric acid (98%), glacial acetic acid (99.5%), sodium hydroxide, sodium chlorite, and glycerol (99.5%) were used as plasticizers for the bio-nanocomposite fabrication and were purchased from SYSTEM-chemAR (Kuala Lumpur, Malaysia).

### 2.2. Cellulose Nanocrystals’ Isolation from Kenaf Fibers

Cellulose nanocrystals from kenaf were prepared by sulfuric acid hydrolysis according to a previously described method [19]. The native cellulose was extracted from kenaf fibers using a chemical treatment. Pure cellulose was obtained from kenaf fibers by chemical extraction with 4% alkali solution at 80–120 °C for 3 h. Bleaching was then carried out using 1.7% sodium chlorite solution and acetic acid buffer solution for 4 h at 80–120 °C. In order to prepare CNCs, cellulose was hydrolyzed in sulfuric acid (65%) at 45 °C for 40 min. The excess sulfuric acid was removed by washing in distilled water for 10 min at 10,000 rpm, by repeated centrifugation. The resulting suspension containing CNCs was dialyzed against distilled water until a constant pH was achieved.

### 2.3. Biocomposite Film Preparation κ-Carrageenan

The κ-carr bionanocomposite films, with the addition of CNCs and OMMT, were fabricated using evaporation casting. A composite with different phases/polarities with an adequate uniform dispersion of hybrid nanoparticles was produced by relying solely on the specific composition and nanoparticle types (CNCs/OMMT) without the addition/involvement of any stabilizers. Step 1: the bionanocomposite film was prepared with single filler loading. The CNCs and OMMT amounts in the composites varied between 2% and 8% (dry weight basis). The film-forming solution containing κ-carr, glycerol, and fillers were prepared using water as a solvent. The solution was heated at 70–90 °C with continuous stirring to obtain a homogeneous gelatinized suspension. The heat was removed and the suspension was stirred continuously for an additional 30 min. A casting knife was used to control the film thickness before it was allowed to dry at room temperature. Step 2: the hybrid bionanocomposite films were then fabricated with the same evaporation casting technique with the hybridization of CNCs and OMMT fillers at the optimum individual nanofiller content based on the tensile test. In order to achieve a homogeneous solution, all film-forming solutions were homogenized for 15 min to prevent particle agglomeration in the matrix phase. The final film thickness was achieved in the 30–50 µm range.

### 2.4. Characterization

The composite films’ mechanical performance was evaluated by measuring the tensile strength and modulus with a universal testing machine (Instron model 5566, Norwood, MA, USA) at room temperature according to the ASTMD882 standard; this is the standard for thin plastic sheeting (film) tensile testing. A 5 mm/min cross speed, 40 mm initial grip distance, and 50 N cell load were used during these tests. The film thicknesses were determined at five points using a digital caliper (Mitotuyo, Kuala Lumpur, Malaysia, ± 0.02 mm). The average value of seven repetitions for each sample was obtained.

The bionanocomposites were characterized by X-ray diffraction to identify nanoclay separation, and nanoparticle and bionanocomposite crystallinity. The diffraction patterns were recorded with radiation (λ = 0.154 nm) generated at 40 kV and 40 MPa, over the 3–50° range.

The dimension and morphology of the kenaf fibers were determined by transmission electron microscopy (TEM) (Philips CM30, North Billerica, MA, USA). A suspension droplet was deposited on a copper grid and covered with a thin carbon film. The CNCs were stained with 2% uranyl acetate solution in deionized water for 1 min and dried at room temperature to enhance the contrast in TEM. The bionanocomposite surfaces were also examined by TEM. The κ-carr bionanocomposite micrographs were obtained from film-forming suspension microdrops cast directly onto the TEM observation grid. The suspension was stained with a 2% uranyl acetate solution for 3 min prior to casting. The cross-section fracture was also studied using field emission scanning electron microscopy (Philips XL-3, North Billerica, MA, USA) (FESEM). The composite films were frozen in liquid nitrogen and broken into small pieces. All samples were coated with gold before observation.

Water absorption for each sample was determined by the weight difference every 5 min. Prior to water exposure, the initial weight (*W*_i_) of the dry sample was measured. Samples were added to 50 mL of distilled water at room temperature and then filtered through Whatman No. 1 filter paper. The final weights of the sample were then measured. Water uptake (%) = (*W*_i_ − *W*_f_) × 100/*W*_i_.

## 3. Results and Discussion

### 3.1. Mechanical Properties

#### 3.1.1. κ-carr/CNC and κ-carr/OMMT Bionanocomposite Films

**Figure 1:**
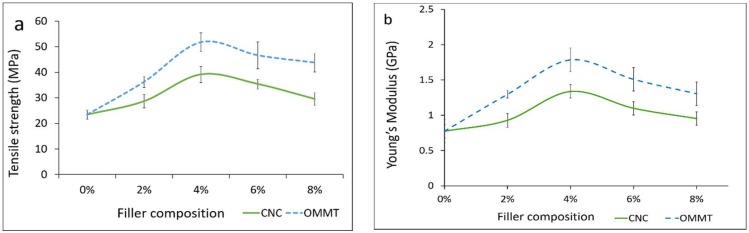
Effects of different types of reinforcement and variations in filler loading on (**a**) tensile strength; and (**b**) Young’s modulus.

The tensile strength of the κ-carr bionanocomposite films with the addition of CNCs (κ-carr/CNCs) and OMMT (κ-carr/OMMT) was determined (Figure 1a). κ-carr/CNCs and κ-carr/OMMT exhibit a higher tensile strength compared with the matrix κ-carr film, which possesses the lowest tensile strength (23.4 MPa). The tensile strength increases with increasing CNCs and OMMT filler loading, with the optimum loading determined to be 4% for each filler type. The κ-carr bionanocomposite film at 4% CNCs and OMMT loading exhibit optimum tensile strengths of 39.1 and 51.8 MPa, respectively. The significant improvement for the κ-carr/CNCs is primarily owing to the CNCs’ high aspect ratio along with the large surface area, resulting in a strong interfacial interaction between the CNC and matrix providing improved reinforcement capability, which relocates the effective stress transfer to the CNC-matrix interface. Moreover, their similar polysaccharide structures form a CNCs homogeneous dispersion in the matrix phase, which is reflected in the strong adhesion between matrix and filler. The significantly improved mechanical properties are attributed to the similarity in chemical structure between κ-carrageenan and CNC, which causes a strong interaction between the κ-carrageenan matrix phases and CNC nanofillers with interfacial hydrogen interaction. This interaction, along with the inherent properties of CNCs, including high surface area and high crystallinity, increase the surface interaction [4,20].

The significant improvement observed for the κ-carr/OMMT bionanocomposite is primarily attributable to κ-carr’s negatively charged poly(disaccharide) units, despite the difference in the matrix and filler polarities. The homogeneously well-dispersed OMMT facilitates interactions that lead to improvements in the bionanocomposite film’s mechanical properties; this is probably due to the OMMT layer’s organic-cationic nature that is located between the platelets and anions at each platelet’s surface. The cations inside the structural gallery play unique roles to stabilize and reinforce by facilitating and strengthening interactions between κ-carr and OMMT. These ionic interactions lead to polymer chain intercalation; intercalation of OMMT and the polymer matrix creates stronger interfacial interactions between the polymer and OMMT surface.

Figure 1b shows the reinforcement type and filler loading effects on the κ-carr/CNCs and κ-carr/OMMT Young’s modulus values. The addition of both CNCs and OMMT causes the Young’s modulus to increase due to the increased stiffness of all composites. This is probably due to the highly crystalline structure of CNCs and OMMT, which increases their stiffness, rigidity, and strength. The 4% κ-carr/CNCs and κ-carr/OMMT display the optimum Young’s modulus at 1337.4 and 1748.5 MPa, respectively. κ-carr/OMMT exhibited the highest stiffness of all filler loading samples compared with the κ-carr/CNCs due to the fact that OMMT itself is intrinsically strong and, therefore, promotes the composite’s higher rigidity. In fact, the layered geometrical shape of OMMT and its high specific area are factors that are often used to explain its reinforcing efficiency in materials involving polymer-chain diffusion into the interlayer OMMT gallery, to achieve the maximum surface area for filler-matrix interactions [21]. The tensile strength and Young’s modulus of both κ-carr/CNCs and κ-carr/OMMT decrease at filler loadings greater than 4%; there is a slight decrease at 6 and 8% filler loadings for CNCs and OMMT, however, it is still higher compared to the pure κ-carr film, especially for the modulus. This behavior can be related to the agglomeration and poor distribution of OMMT and CNCs inside the matrix; hence, their performance as a reinforcement in the matrix depends on the loading level. The reduction of tensile strength suggested that the stress was not uniformly distributed above optimum filler loadings, leading to less efficient stress transfer between the nanoparticles and the matrix. Other investigations have also reported a similar behavior for the decrease in tensile strength and modulus with cellulose nanofibril (CNF) incorporation into an alginate biopolymer using solution casting, due to CNF aggregation, as observed by scanning electron microscopy (SEM) [22]. Similar behavior was observed in reduction of the tensile strength and modulus with a higher nanoclay content for alginate nanocomposite films [23].

#### 3.1.2. Hybrid κ-carr/CNCs/OMMT Bionanocomposite Films

**Figure 2:**
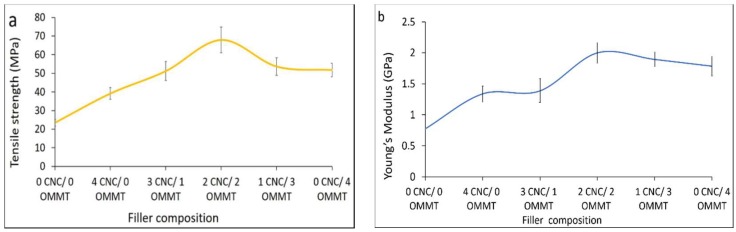
Cellulose nanocrystal (CNC) and organically modified montmorillonite (OMMT) hybridization effects on (**a**) tensile strengths; and (**b**) Young’s modulus of hybrid bionanocomposites.

Figure 2a shows how the tensile strength affects various ratios of hybridized CNCs and OMMT at a 4% filler loading. The tensile strength of the hybrid κ-carr/CNCs/OMMT bionanocomposite increases for all hybrid bionanocomposites when compared with the κ-carr/CNCs and κ-carr/OMMT at the identical 4% filler loading. The κ-carr/CNCs/OMMT bionanocomposite exhibits a synergistic effect on the mechanical properties that leads to higher tensile strength values (67.9 MPa) with a filler loading of equal ratio of CNCs and OMMT (1:1) compared with the κ-carr/CNCs (39.1 MPa) and κ-carr/OMMT (51.8 MPa) with an identical filler loading. Moreover, the other hybrid bionanocomposites with different CNCs/OMMT ratios also exhibit improvements over those reinforced with individual fillers, which is due to the compatibility between the κ-carr matrix with CNCs and OMMT, despite the difference in polarity. This enhancement is also related to a CNC particle network formation and the incorporation of OMMT particles with different dimensions which occupy the space in the matrix phase; this provides an improved reinforcement capability in the hybrid κ-carr/CNCs/OMMT system because of a high specific area, more interfacial interaction, and efficient stress transfer which supports high levels of transmitted stress.

Figure 2b reveals that the Young’s modulus was highest, at the optimum ratio of 1:1 for CNCs:OMMT. Young’s modulus values increased for all hybrid κ-carr/CNCs/OMMT samples, owing to the improved uniform distribution, which is strongly related to the dimensions and relatively high CNCs and OMMT particle aspect ratio. Hybrid κ-carr/CNCs/OMMT exhibits a higher stiffness which is probably attributed to the high rigidity, high crystallinity, together with their good affinities through different interactions in one hybrid system, resulting from the good dispersion of CNCs and OMMT in the matrix phase. Hybrid κ-carr/CNCs/OMMT with (1 CNCs:3 OMMT) exhibits a higher mechanical strength compared with the (3 CNCs:1 OMMT) because of the small OMMT amount in the composite which could restrict the polymer-matrix deformation through improved interaction between the matrix and filler. The κ-carr and OMMT form an interfacial ionic interaction and the matrix and filler intercalate, indicating that they are directly involved in the association between the carrageenan and OMMT surfaces. Hybridization involving the filler enables the enhanced production of super-stiff bionanocomposite films. The addition of OMMT enables cationic exchange in the gallery between the platelets, with polyelectrolyte behavior exhibited by polysaccharide polymers like κ-carrageenan (polyanion). The κ-carrageenan has one negative charge per disaccharide unit. The main role of inorganic cations in the gallery is to act as a bridge for interaction between the two polyanionic components of polyanion κ-carrageenan and the negative charges present in the platelets and clay surface. The polymer chain intercalation enables ionic interaction between the matrix and reinforcement. A similar observation was reported by Franchi et al. [24].

### 3.2. X-ray Diffraction Studies of the Bionanocomposite Thin Films

**Figure 3:**
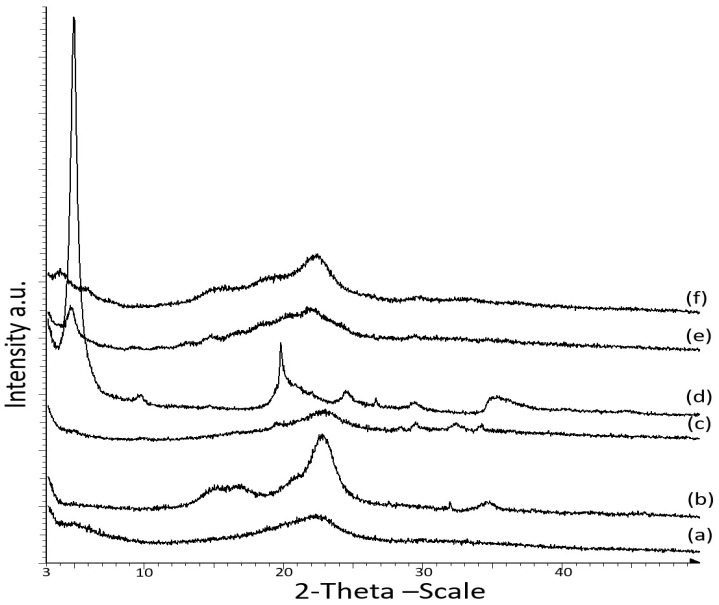
X-ray diffraction (XRD) patterns of (**a**) matrix κ-carrageenan; (**b**) CNC powder; (**c**) κ-carr/CNC bionanocomposite film; (**d**) OMMT powder; (**e**) κ-carr/OMMT bionanocomposite film; and (**f**) hybrid κ-carr/CNC/OMMT bionanocomposite film.

Figure 3 displays the X-ray diffraction (XRD) patterns of the CNCs and OMMT nanoparticles and the κ-carr-based bionanocomposite at the optimum 4% filler loading. XRD was performed to analyze the OMMT dispersion in the polymer matrix and the structural properties of the OMMT and CNCs-reinforced κ-carr film. Crystalline CNCs exhibit an XRD peak at 2θ = 22.5°, while that for the matrix κ-carr film is at 2θ = 22°. The XRD pattern observed after the addition of CNCs to the polymer matrix reveals broad peaks at 2θ = 22.5° and small peaks at 2θ values of 29.5°, 32.5°, and 34°. According to the specific amount of CNCs loading (4%), the peak at 2θ = 22.5° clearly exhibits sharpness, which reveals that the CNCs retain crystallinity in the composite film.

However, OMMT exhibits a characteristic crystallinity peak at 2θ = 4.86° (*d*_001_ = 1.86 nm). Intercalation/exfoliation causes the diffraction peak to shift toward a lower angle [25]. The 4% OMMT addition in the polymer matrix resulted in polymer chain intercalation between the OMMT interlayers, which increased the interlayer spacing, leading to a shift in the diffraction peak to a lower 2θ value of 4.82° (*d*_001_ = 1.91 nm). Only a small increase in the clay *d*_001_ from 1.86 to 1.91 nm was achieved since κ-carr and OMMT possess different affinities, rendering it easily intercalated into the OMMT interlayer spacing. Previous research has demonstrated that the intercalation of biopolymer cassava starch in silicate layer montmorillonite (MMT) occurred at 17.62 Å (1.76 nm) for a nanocomposite film at 4 wt% of montmorillonite [26]. Another investigation has also reported a similar pattern for the interlayer distance of a layered silicate of the intercalated biopolymer, chitosan, a natural cationic polysaccharide, intercalated into MMT by an ion-exchange reaction at 1.45 nm (monolayer) or 2.09 nm (bilayer) depending on the chitosan chains’ adsorption number [27]. The XRD pattern of Na^+^-MMT intercalation with cationic functional starch showed basal *d*_001_-spacing (1.26–1.44 nm) [28]. The low-cost powder bionanocomposite based on cationic starch (CS) and Na-montmorillonite (MMT) exhibits interlayer spacing at 1.84 nm in the clay structure due to the intercalation of polysaccharide molecules between silicate layers [29]. These results agreed well with previous studies, wherein XRD equipment was used to analyze the structure of the nanoclay particles in different polymer matrices. XRD patterns showed that intercalation occurred, with a diffraction peak shift to a lower angle, accompanied by broadening and intensity decrease in the diffraction peak. This indicated a disordered intercalated/exfoliated structure [5,24,30,31].

These interesting examples of layered silicate montmorillonite with natural biopolymers are promising for improving the reinforcing effect in applications for enhanced performance. However, the strategy of using organo-modified montmorillonite was chosen because of the higher interlayer spacing, or d-spacing (*d*_001_), of 1.8 nm for OMMT itself; due to cationic exchange insertion between the platelets, the polymer chains can diffuse into the interlayer gallery. Polysaccharides exhibit a polyelectrolyte behavior and, similar to carrageenan, are primarily polyanions. The formation of a physical network, stabilized by ionic interaction between OMMT and the κ-carr chains through cationic exchange, occurs because of the basal spacing, *d*_001_, leading to a good interfacial interaction. Even with a slight increase in basal spacing, intercalation occurs between κ-carr/OMMT. However, OMMT hybridization with CNCs in the polymer resulted in an OMMT broad peak at a lower 2*θ* value (3.85°) for the composite film due to the expansion in the interlayer spacing (*d*_001_ = 2.29 nm).

A previous study by Silvestre et al. [32], demonstrated that intercalated nanocomposites are normally interlayered by a few polymer chains between the nanolayer structure interlayer galleries. When clay minerals interact with biopolymers, such as organically modified montmorillonite, adsorption is also influenced by the nature of the exchangeable cations. The unique OMMT intercalation behavior in the κ-carr biopolymers and the interactions between OMMT and the carrageenan structural components is a result of its fine particle size, large surface area, and specific charge characteristics [24], which are responsible for rendering OMMT a very important and powerful biopolymer reinforcing filler. Usually, a well-developed polymer/OMMT nanocomposite yields improved mechanical strength compared with the matrix polymer since the uniformly dispersed nanosized OMMT particles provide a high interfacial area and ionic bonds between the nanoclay and matrix polymer [33].

### 3.3. Morphological Characterization

#### 3.3.1. Transmission Electron Microscopy of Cellulose Nanoparticles

**Figure 4:**
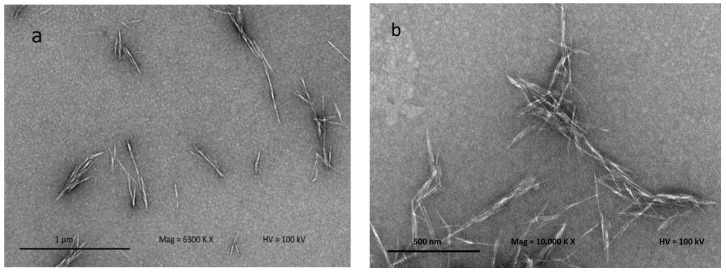
Transmission electron microscopy (TEM) micrographs of hydrolyzed kenaf under different magnifications: (**a**) 6300× and (**b**) 10,000×.

Figure 4 shows the TEM micrograph to confirm the separation of individual crystalline nanoparticles (CNCs) produced after acid hydrolysis. Acid hydrolysis disintegrates the cellulose and dissolves the lateral-order amorphous domain, leaving only the highly crystalline domain. This process also facilitates cellulose fiber defibrillation on a nanoscale level. CNC structures can be clearly observed using TEM because the CNCs allow a quite stable dispersion in water. CNCs obtained from kenaf fiber result in needle-like shapes, similar to the observation of Johar and Ahmad [20]. The CNCs produced possess dimensions between 12–15 nm in diameter and 101–250 nm in length. The CNC particles produced on the nanoscale possess a high aspect ratio; it is well known that the CNC aspect ratio plays a crucial role in their reinforcing capability. The image displays some lateral agglomeration which can be clearly observed among nanoparticles; this is probably due to high concentrations of the CNC in suspension, and causes self-association among the particles via hydrogen bonding of the CNC particles [34].

#### 3.3.2. Scanning Electron Microscopy Study of Bionanocomposites

**Figure 5:**
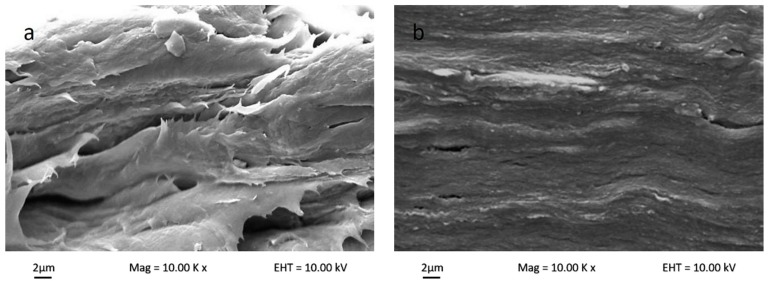

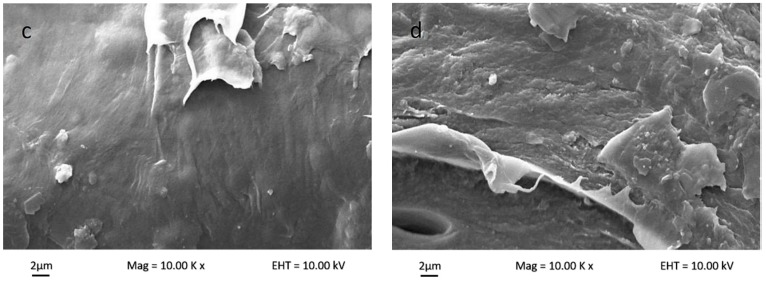
Surface cross-section scanning electron microscopy (SEM) micrographs of (**a**) κ-carr matrix film; (**b**) κ-carr/4% CNC bionanocomposite; (**c**) κ-carr/4% OMMT bionanocomposite; and (**d**) hybrid κ-carr/4% (CNC/OMMT) (1:1) bionanocomposite.

Figure 5 shows the polymer matrix cross-sectional SEM micrograph for bio-nanocomposite κ-carr/CNC, bio-nanocomposite κ-carr/OMMT, and hybrid bio-nanocomposite κ-carr/CNC/OMMT. The uniform nanofiller dispersion and good interfacial bonding between the filler and polymer matrix, which are correlated to the morphologies with the composite fracture, explains the changes in the mechanical properties. The original polymer matrix film possesses some smooth surfaces, which structurally indicates that the sample can easily fracture. The internal morphology changes substantially with the addition of the nanoparticle CNCs and OMMT in the polymer at optimum filler loading (4%). The addition of CNCs yields a compact structure with homogeneous CNC dispersion in the polymer matrix. The morphological structure indicates that good CNC dispersion enables a strong interaction between CNCs and κ-carr. The similarity in chemical structure between CNCs and κ-carr ensure good reinforcement compatibility, and the structure is stabilized laterally with the hydrogen bond formation between hydroxyl groups. The κ-carr with OMMT addition exhibits a morphology with no significant pores and cracks visible, indicating that there is compatibility and interfacial contact between OMMT and polymer κ-carr. The 4% OMMT addition embrittles the composite product; however, the cross-sectional image shows a smooth surface with a higher mechanical strength. However, the hybrid composite sample appears to possess the combined effects of CNC and OMMT. The composite film exhibits a compact structure with cracks. The cross-section surface appears rough and homogeneous. Thus, hybridization and combination enhanced stiffness with different reinforcement sizes.

#### 3.3.3. Transmission Electron Microscopy Study of Bionanocomposite

**Figure 6:**
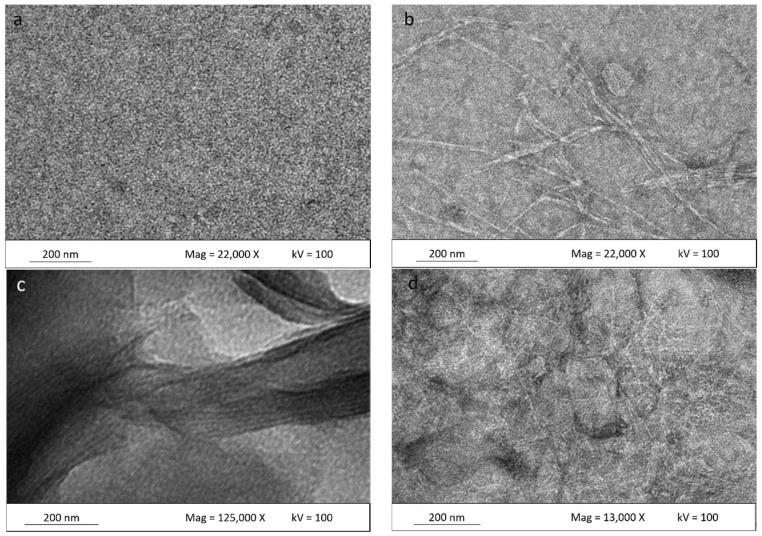
Surface TEM micrographs of (**a**) κ-carr matrix film; (**b**) κ-carr/4% CNC bionanocomposite; (**c**) κ-carr/4% OMMT bionanocomposite; and (**d**) hybrid κ-carr/ 4% (CNC/OMMT) (1:1) bionanocomposite.

TEM micrographs of the matrix polymer and κ-carr/CNCs, κ-carr/OMMT, and hybrid κ-carr/CNCs/OMMT bionanocomposite films, with 4% filler loadings, are shown in Figure 6. A smoothly dispersed matrix is clearly seen in Figure 6a, whereas uniform CNC dispersion in the matrix can be seen in Figure 6b. The CNC particles form a network in the carrageenan matrix, as compared with the single CNC distribution in a water-based system. This is in good agreement with the excellent mechanical properties observed due to the CNCs’ ability to restrict matrix mobility, thereby contributing to improving the material properties. A similar dispersion pattern involving nanocellulose in starch also has been reported [35].

Figure 6c depicts an enlarged view of OMMT particles in the κ-carr/OMMT polymer matrix, confirming that the OMMT is intercalated. The spacings between the platelets were determined by XRD (d-spacing). Intercalated structures are formed when polymer chains are intercalated between the OMMT layers, resulting in a well-ordered multilayer structure of alternating polymer matrix and OMMT layers with a repeat distance between them [5]. Haq et al. [36] and Botana et al. [4], show a similar micrograph of an intercalated clay platelet gallery. However, Figure 6d shows a hybrid bio-nanocomposite CNCs/OMMT in the matrix polymer. It is very difficult to definitively say whether the OMMT distributes well. However, Tang et al. [37] have reported on an image of clay in starch; they display the same distribution pattern of clay with the OMMT in the hybrid composite. They reported that a multilayered nanostructure exists and some single clay platelets are distributed at the image edge. There are more particles packed together in between the CNC network. According to the XRD pattern (Figure 3), the OMMTs have higher spacing between the platelets. The same image shows that CNC has an improved uniform network in the matrix along with the OMMT particles. These observations are very relevant because they could provide an improved understanding of the CNC and OMMT barrier effect based on the presence of a network structure and intercalated OMMT, which reduce solubility to a significant extent and, hence, improve the mechanical properties. This demonstrates that the CNC-OMMT combination that exhibits synergistic effects will possess the benefits of each constituent, resulting in a good stiffness toughness balance.

### 3.4. Photographic Images of Bionanocomposites

**Figure 7:**
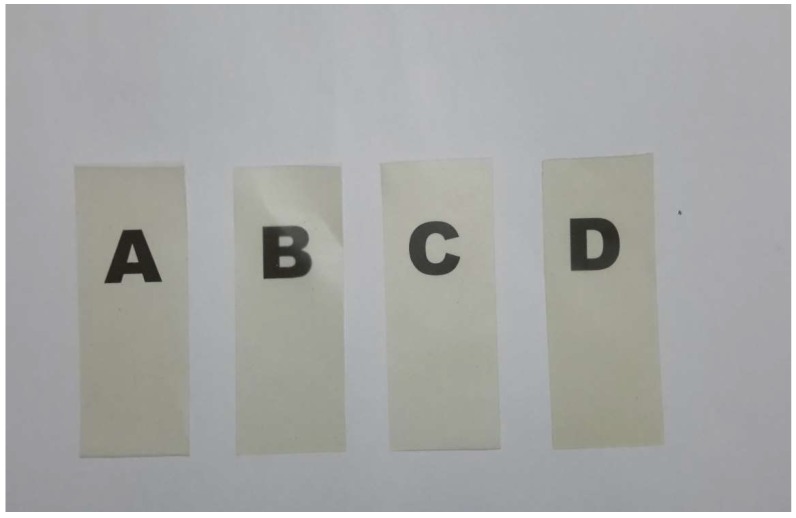
Typical photographic images of 30 μm-thick films of (**a**) κ-carr matrix; (**b**) κ-carr/CNC bionanocomposite; (**c**) κ-carr/OMMT bionanocomposite; and (**d**) hybrid κ-carr/CNC/OMMT bionanocomposite.

Figure 7 shows typical photographic images of the carrageenan matrix film and its bionanocomposites. Each sample’s contact transparency was evaluated; the samples appear to exhibit similar behaviors. Samples containing CNCs exhibit the best optical properties, while the samples containing OMMT are less transparent. Since the contact transparency of the films containing CNCs exhibit better transparencies, the CNCs must be better dispersed in the film than OMMT, which scatters light significantly. This also indicates the well-dispersed nature of OMMT in the polymer matrix. Tiny CNCs and OMMT particles in the κ-carr matrix disperse well to prevent light-scattering significantly. The hybrid bionanocomposite also displays better transparency, together with (b) and (c), which is consistent with the observation that the hybridized CNCs/OMMT nanofillers are homogeneously well-dispersed in the polymer matrix.

### 3.5. Water Uptake of Bionanocomposites

**Figure 8:**
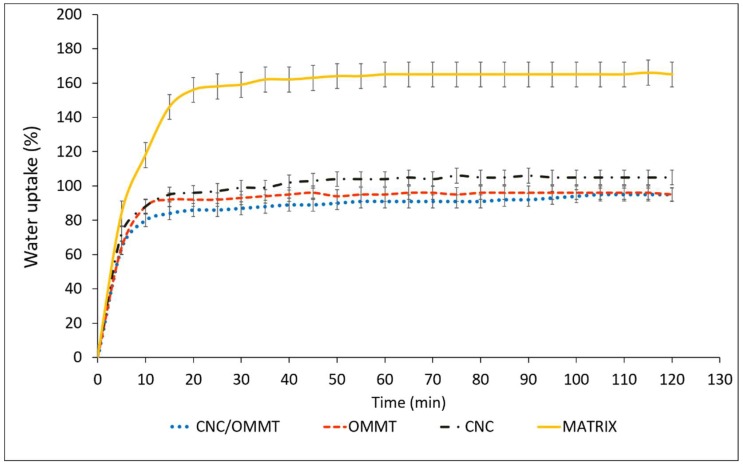
Water uptake of κ-carr matrix films: κ-carr matrix, κ-carr/CNC bionanocomposite, κ-carr/OMMT bionanocomposite, and hybrid κ-carr/CNC/OMMT bionanocomposite.

Figure 8 shows the effects of optimum filler loading (4%) of OMMT and CNCs effects on water uptake of the κ-carr-based film. Reducing biopolymer water sensitivity and enhancing its water resistance are significantly important because the bionanocomposite performance is enhanced. CNC incorporation significantly reduces the κ-carr-based films’ water uptake percentage. After 2 h immersion in water, the water uptake of the control κ-carr film was 166%. The incorporation of CNCs at 4% filler loading leads to a reduction in water uptake to only 105%. Haq et al. [36] have also reported that a low nanocellulose content led to slower diffusion processes as evidenced by the composite films’ increased tortuosities. Even though both the κ-carr matrix and CNCs are considered to be hydrophilic, the barrier to water permeability is enhanced due to the high crystallinity of the CNCs filler added to the bionanocomposite and is related to strong hydrogen bonding between the hydroxyl groups of the nanoparticles and κ-carr [33]. These interactions improve the cohesiveness of the κ-carr polymer matrix and enhance its resistance to water, as water molecules are not able to sufficiently break these strong bonds [28]. The results demonstrate that nanocellulose efficiently improves barrier properties through network formation [Figure 6b] in the polymer matrix that can reduce water absorption.

Meanwhile, the water uptake of the OMMT-reinforced composite film (4%) decreases by about 68% over the control matrix. One of the most significant effects of OMMT on the polymer matrix properties is its ability to dramatically improve the polymer barrier properties. OMMT sheets are naturally impermeable; they create a maze of tortuous paths that retards water molecule diffusion through the polymer matrix. OMMT addition results in a nanocomposite that is more hydrophobic in nature. Reinforcement by the hybridization of CNCs and OMMT results in a significant decrease in water uptake, by about 74% at 4% filler loading, compared with the matrix without the filler. This is primarily attributed to the higher tortuosity created by the incorporation of the CNCs network and the distribution of intercalated OMMT in the matrix; both fillers will appropriate the free volume within the polymer matrix which inhibits water molecule diffusion through the nanocomposite, Henriette de Azeredo [7], as discussed previously (Figure 6d). In this way, the hybridization of CNCs and OMMT reveals a high potential for overcoming the limitations of κ-carr biopolymer film. The diffusion coefficient largely depends on the nature of the polymer and morphology of the bionanocomposite material produced.

## 4. Conclusions

CNCs and OMMT were added to κ-carrageenan biopolymer to compare the properties of bionanocomposites reinforced with different organic and inorganic nanofillers. Results showed that the nanofiller content affected the properties of the nanocomposites. The reinforcing effect of the hybridization of both fillers was investigated for various ratios at the optimum filler loading. The hybrid bionanocomposite film exhibited synergistic mechanical properties compared with the nanocomposite film reinforced with individual nanofillers. These synergistic effects were primarily due to the higher crystallinities of both CNCs and OMMT, large specific area, and high aspect ratio with different dimensions, which restricted the matrix mobility, and improved the reinforcement, stiffness, and rigidity. The water resistance of the bionanocomposites was drastically affected with different filler types and hybridization. Both CNCs and OMMT decreased the water uptake of the nanocomposite. Reinforcement by the hybridization of CNCs and OMMT resulted in a significantly decreased water uptake due to the increase in diffusion path tortuosity. SEM images of the sample also revealed internal morphology changes. Regardless of its different polarity, OMMT was compatible with the κ-carr biopolymer, and no phase separation was observed, due to the unique properties of both OMMT and κ-carr that enabled stabilizing ionic interactions.
